# Supplementary material for: ThermoTargetMiner as a proteome integral solubility alteration target database for prospective drugs against lung cancer
Source: Commun Chem. 2026 Oct 2;9:312. doi: 10.1038/s42004-026-02207-z (PMC13633240; doi:10.1038/s42004-026-02207-z)
Supplement: Supplementary file 1 [file 42004_2026_2207_MOESM1_ESM.pdf]

nature portfolio

Peer Review File

ThermoTargetMiner as a proteome integral solubility alteration target database for prospective drugs against lung cancer

Corresponding Author: Professor Roman Zubarev

This manuscript has been previously reviewed at another journal. This document only contains information relating to versions considered at Communications Chemistry.

This file contains all reviewer reports in order by version, followed by all author rebuttals in order by version.

Version 0:

Reviewer comments:

Reviewer #1

(Remarks to the Author)
The authors addressed all the raised concerns at my side.

Reviewer #3

(Remarks to the Author)
The revised manuscript is substantially improved and presents the study in a clearer and more convincing manner. The authors have addressed my major concerns regarding data presentation, interpretation, and the proportionality of conclusions.

Overall, I consider the revision satisfactory. While some limitations remain inherent to the experimental design, these are now more appropriately acknowledged and no longer undermine the central conclusions of the manuscript.

Reviewer #4

(Remarks to the Author)
I co-reviewed this manuscript with one of the reviewers who provided the listed reports. This is part of the Communications Chemistry initiative to facilitate training in peer review and to provide appropriate recognition for Early Career Researchers who co-review manuscripts.

Reviewer #5

(Remarks to the Author)
The authors have addressed most of my comments.
The only comment that was not satisfactorily addressed was my comment 4: “In the analysis of pro-targets, it would be interesting to have a more general analysis of all drugs and their known targets (what is described in Table 2). How many known targets are detected at the different levels of k? And how many are missed, and how many false positives are found at lower levels of k?” The authors write that they have expanded the discussion, but I do not find any text related to this. I think it would be more adequate to perform the analysis and add it in the results section. A simple bar chart with number of true positives, false positives and false negatives for k=1, 2, 3, 4 would be sufficient.

Reviewer #6

(Remarks to the Author)
I co-reviewed this manuscript with one of the reviewers who provided the listed reports. This is part of the Communications Chemistry initiative to facilitate training in peer review and to provide appropriate recognition for Early Career Researchers

who co-review manuscripts.

Version 1:

Reviewer comments:

Reviewer #5

(Remarks to the Author)
The authors have now addressed all of my comments.

Reviewer #6

(Remarks to the Author)
I co-reviewed this manuscript with one of the reviewers who provided the listed reports. This is part of the Communications Chemistry initiative to facilitate training in peer review and to provide appropriate recognition for Early Career Researchers who co-review manuscripts.

Open Access This Peer Review File is licensed under a Creative Commons Attribution 4.0 International License, which permits use, sharing, adaptation, distribution and reproduction in any medium or format, as long as you give appropriate credit to the original author(s) and the source, provide a link to the Creative Commons license, and indicate if changes were made.
In cases where reviewers are anonymous, credit should be given to 'Anonymous Referee' and the source.
The images or other third party material in this Peer Review File are included in the article's Creative Commons license, unless indicated otherwise in a credit line to the material. If material is not included in the article's Creative Commons license and your intended use is not permitted by statutory regulation or exceeds the permitted use, you will need to obtain permission directly from the copyright holder.
To view a copy of this license, visit https://creativecommons.org/licenses/by/4.0/

We sincerely thank all reviewers for their careful evaluation of our manuscript and for their constructive and insightful comments. We greatly appreciate the time and effort invested in reviewing our work, which have helped us improve the clarity, rigor, and overall presentation of the manuscript.

We are encouraged that the reviewers recognized the improvements made during revision and found that our responses addressed the major concerns regarding data presentation, interpretation, and the scope of our conclusions. We have carefully considered all comments and have further revised the manuscript accordingly. In particular, following the suggestion from Reviewer #5, we have performed an additional systematic evaluation of pro-target identification across different k values using known drug targets from DrugBank. This analysis has now been added to the Results section and Supplementary Figure 4, providing a quantitative assessment of true positives, false positives, and false negatives to better justify the selection of k = 2 as the criterion for defining pro-targets.

We would like to clarify that the “true targets” used in this benchmarking analysis were defined based on targets annotated in DrugBank rather than all experimentally reported targets from the literature. Therefore, the number of true positives may be underestimated, particularly for kinase inhibitors, for which some biologically relevant kinase targets are not currently included in DrugBank. Similarly, targets classified as false positives in this analysis should not necessarily be considered confirmed false positives, as some of them may represent potential novel targets or previously unannotated drug-target interactions.

Nevertheless, this analysis provides a relative evaluation of different k values. When k = 1, the proportion of predicted targets classified as false positives was substantially higher, indicating a lower stringency and a higher likelihood of including nonspecific candidates. Increasing the threshold to k = 2 markedly reduced the number of such candidates while retaining a considerable number of known targets. In contrast, using k = 3 resulted in a substantial reduction in the number of identified pro-targets, potentially leading to the loss of relevant targets. Therefore, considering the balance between target recovery and candidate specificity, we selected k = 2 as the optimal criterion for defining pro-targets.
