# Supplementary material for: ThermoTargetMiner as a proteome integral solubility alteration target database for prospective drugs against lung cancer
Source: Commun Chem. 2026 Oct 2;9:312. doi: 10.1038/s42004-026-02207-z (PMC13633240; doi:10.1038/s42004-026-02207-z)
Supplement: Supplementary file 2 [file 42004_2026_2207_MOESM2_ESM.pdf]

SUPPLEMENTARY INFORMATION

ThermoTargetMiner as a proteome integral solubility alteration target database for prospective drugs against lung cancer

Lyu et al.

Supplementary Table 1. Assignment of the TMT channels in the experiments 1-5 for each of the cell lines (A549 and NCI-H82) and analysis type (in intact cells and lysate).

	**Experiment 1**	**Experiment 2**	**Experiment 3**	**Experiment 4**	**Experiment 5**
**TMT126**	DMSO	DMSO	DMSO	DMSO	DMSO
**TMT127N**	Methotrexate	Methotrexate	Methotrexate	Methotrexate	Methotrexate
**TMT127C**	Crizotinib	AZD5363	RO4929097	Sunitinib	Brigatinib
**TMT128N**	Docetaxel	Obatoclax Mesylate	KOS-862	Prinomastat	Defactinib
**TMT128C**	Ponatinib	Gossypol	AZD1775	Phenethyl isothiocyanate	Bexarotene
**TMT129N**	Sorafenib	Dabrafenib	L-alanosine	Metformin	Selumetinib
**TMT129C**	Gefitinib	TAK-931	Prexasertib	Dichloroacetate	Napabucasin
**TMT130N**	Lapatinib	Seliciclib	Guadecitabine	Anlotinib	Desipramine
**TMT130C**	Pazopanib	Navarixin	Itacitinib	Lonafarnib	7-hydroxystaurosporine
**TMT131N**	Ruxolitinib	Regorafenib	Trametinib	Pirfenidone	Etalocib
**TMT131C**	Apatinib	Vorinostat	Pevonedistat	Exisulind	Binimetinib
**TMT132N**	Cabozantinib	Ganetespib	Entrectinib	Cilengitide	Alisertib
**TMT132C**	Fludarabine	Everolimus	YM155	Dihydroartemisinin	Palbociclib
**TMT133N**	Topotecan	Olaparib	Berzosertib	Iniparib	Empty
**TMT133C**	Epirubicin	Pictilisib	Acalabrutinib	Imatinib	Empty
**TMT134**	SDS	Pioglitazone	Salirasib	ZD4054	Empty

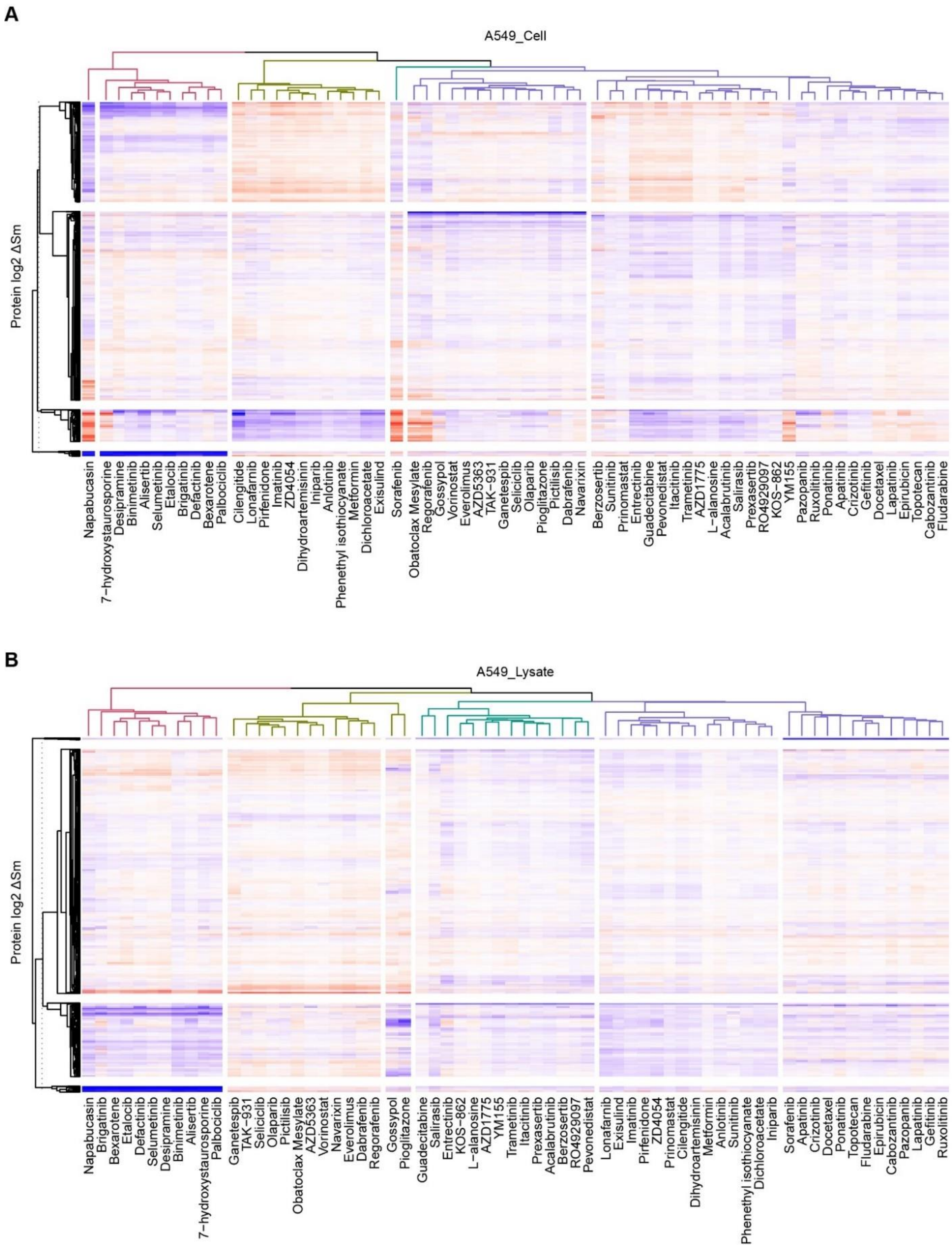

Supplementary Figure 1. Heatmap based on clustering of log2 transformed PISA fold changes for proteins in the A549 lysate dataset (**A**) and A549 intact cell dataset (**B**).

A

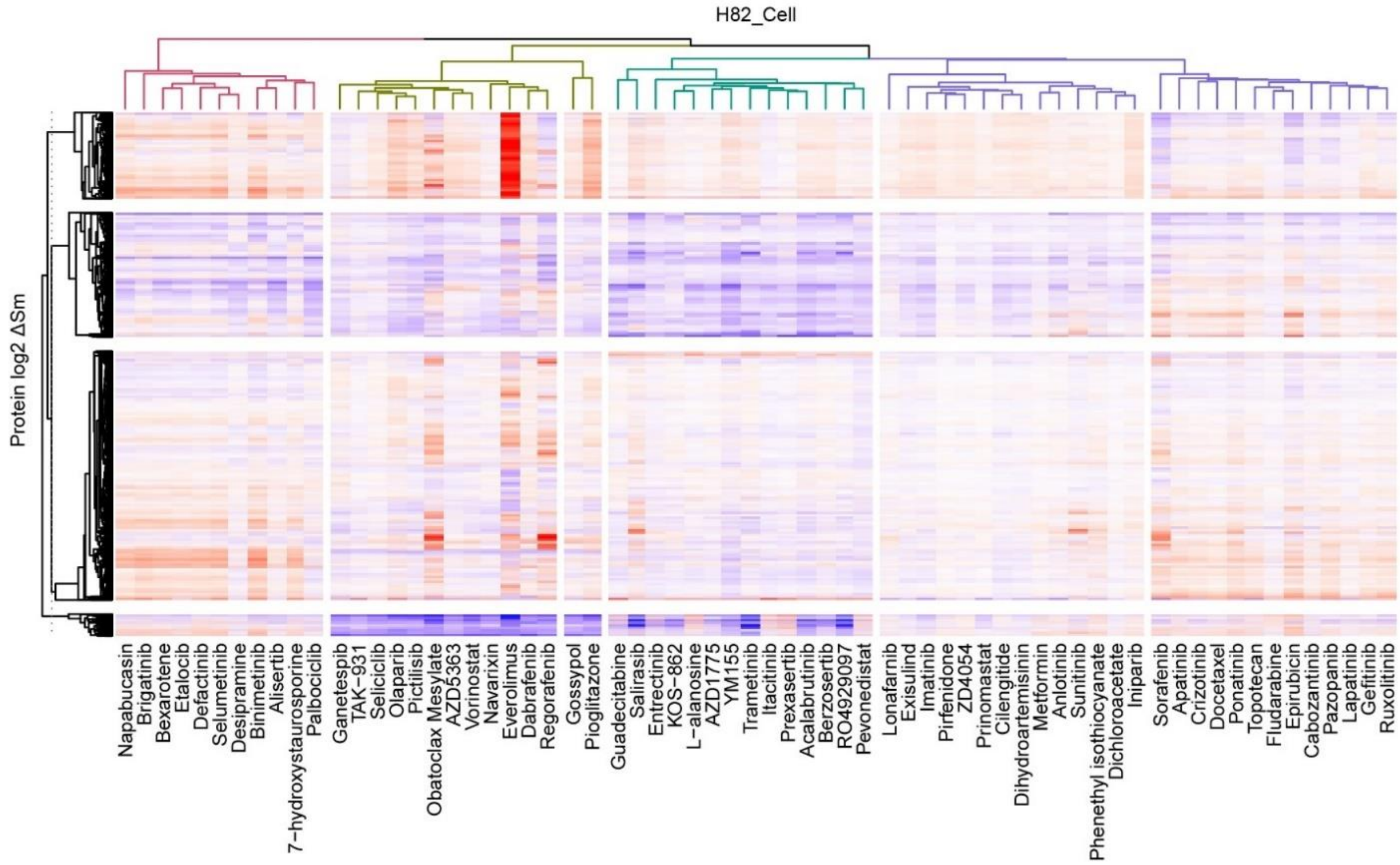

B

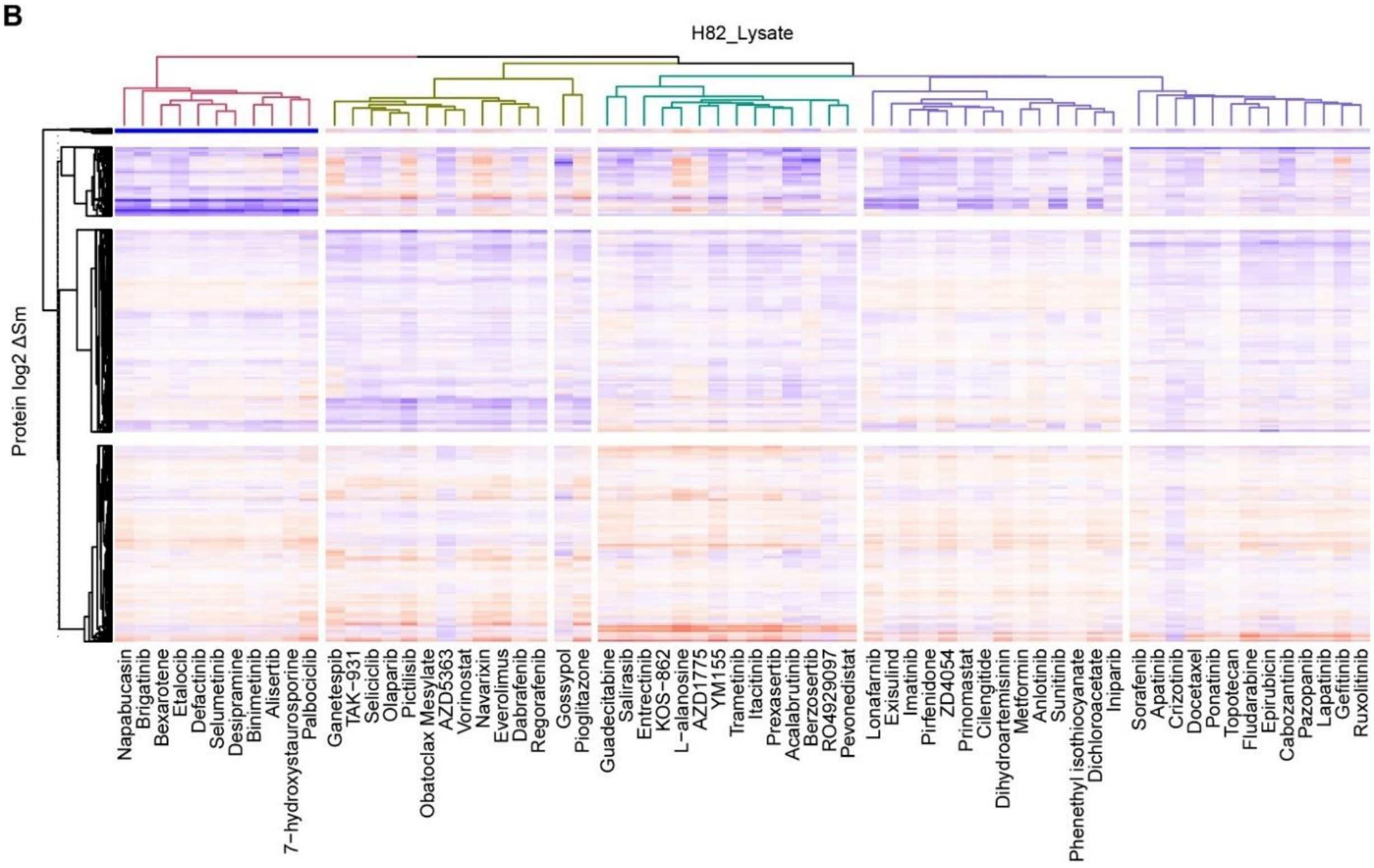

Supplementary Figure 2. Heatmap based on clustering of log2 transformed PISA fold changes for proteins in the H82 lysate dataset (**A**) and H82 intact cell dataset (**B**).

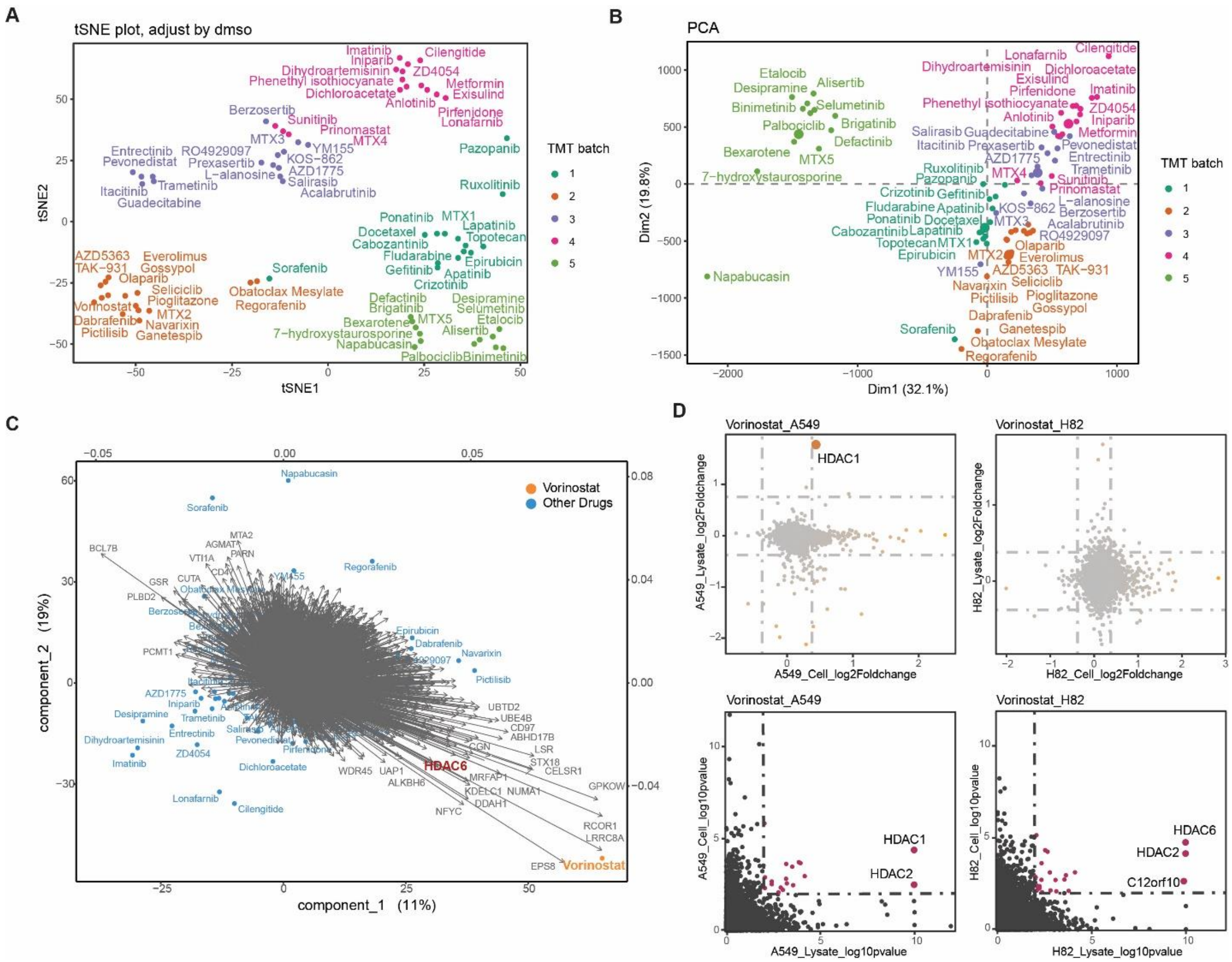

Supplementary Figure 3. Comparison of statistical analysis approaches for ThermoTargetMiner data. (A-B) Unsupervised clustering reveals dominant batch effects. (A) t-SNE analysis of A549 PISA data. Despite normalizing drug-treated samples against batch-matched DMSO controls (see Methods), samples cluster primarily by TMT experimental batch (indicated by color) rather than drug mechanism. **(B)** Principal Component Analysis (PCA) of sum- and median-normalized data. Similar to t-SNE, the variance is driven by batch effects ("Set") rather than biological differences, obscuring specific drug-target signals. **(C) Limitations of one-vs-all PLS-DA.** A biplot visualizing a PLS-DA model comparing one specific drug (e.g., vorinostat) against all other drugs. While this approach can identify true targets (e.g., HDACs), it is suboptimal due to projection biases requiring manual adjustment. **(D) OPLS-DA improves target identification sensitivity.** Comparison of target detection using standard thresholding versus OPLS-DA. **Upper panels:** Scatter plots using standard filtering criteria (Fold Change > 1.3 and p-value < 0.05). This method shows low sensitivity; for example, only HDAC1 is identified in A549 lysate/cell data, and no targets passed thresholds in H82 data. **Lower panels:** Application of OPLS-DA scoring and coordinates transformation to the same dataset. This method significantly enhances signal-to-noise separation, successfully identifying known targets (HDAC1, HDAC2, and HDAC6) as top hits.

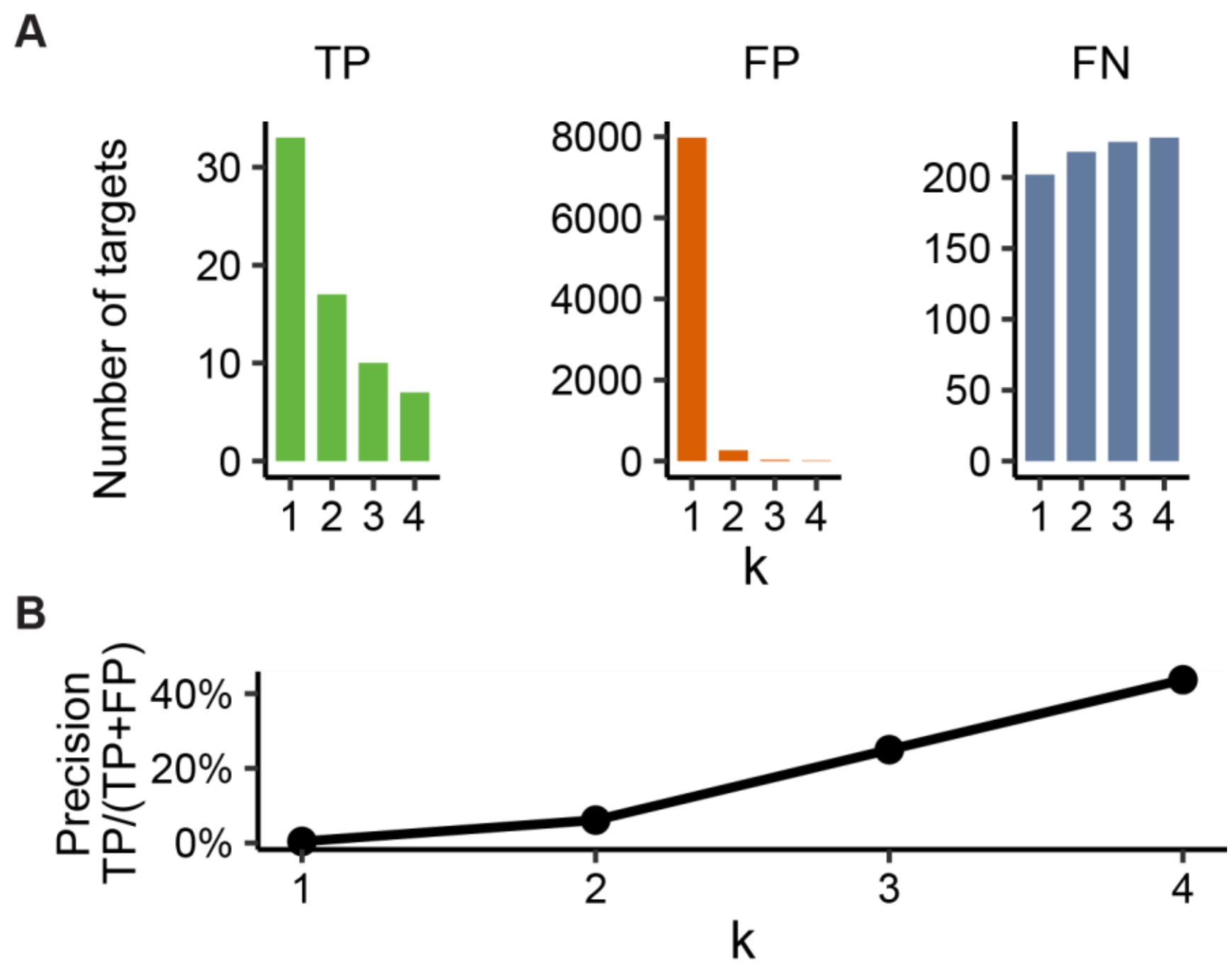

Supplementary Figure 4. Performance of pro-target identification across different reproducibility thresholds (k). Numbers of true positives (TP), false positives (FP), and false negatives (FN) are shown for k = 1-4 based on comparison with DrugBank annotated targets (**A**). Increasing k reduces false positives while increasing missed targets, resulting in higher precision (TP / (TP + FP)) but lower target recovery (**B**). Only drugs with annotated targets were included.

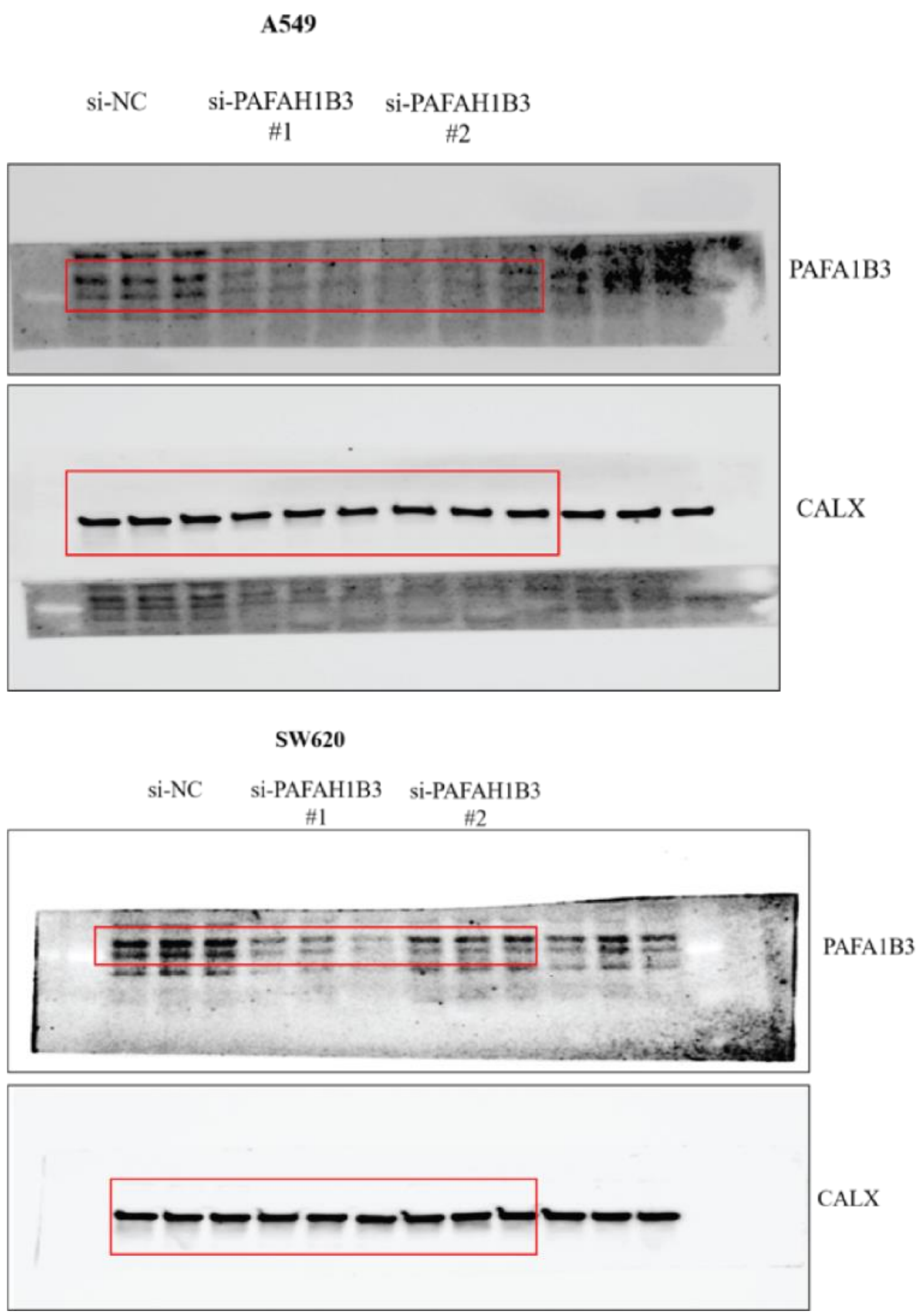

Supplementary Figure 5. Uncropped blot corresponding to Figure 6D. Uncropped and unprocessed immunoblots showing PAFAH1B3 and calnexin in A549 and SW620 cells transfected with a negative control (NC) siRNA or two different siRNAs targeting PAFAH1B3, followed by treatment with PEITC for 72 h. DMSO-treated cells were included as vehicle controls for each transfection condition. Calnexin was used as a loading control. The full-length blot images corresponding to the cropped immunoblots presented in Figure 6D are shown.
