# Supplementary material for: ThermoTargetMiner as a proteome integral solubility alteration target database for prospective drugs against lung cancer
Source: Commun Chem. 2026 Oct 2;9:312. doi: 10.1038/s42004-026-02207-z (PMC13633240; doi:10.1038/s42004-026-02207-z)
Supplement: Supplementary file 3 [file 42004_2026_2207_MOESM3_ESM.pdf]

Description of Additional Supplementary File

File name: Supplementary Data 1
Description: Protein-level quantitative data from PISA experiments, including normalized fold changes and p-values for all detected proteins across compounds and experimental conditions.

File name: Supplementary Data 2
Description: OPLS-DA-derived protein coordinates and significance estimates used for multivariate target prioritization and identification of pro-targets.
